# Outcomes of Modular Knee Arthrodesis for Challenging Periprosthetic Joint Infections

**DOI:** 10.1016/j.artd.2021.10.015

**Published:** 2022-01-22

**Authors:** Alexandra I. Stavrakis, Erik N. Mayer, Sai K. Devana, Madhav Chowdhry, Matthew V. Dipane, Edward J. McPherson

**Affiliations:** Department of Orthopaedic Surgery, University of California Los Angeles, Los Angeles, CA, USA

**Keywords:** Knee fusion, Periprosthetic joint infection

## Abstract

**Background:**

Modular knee arthrodesis (MKA) is a salvage treatment option for patients with challenging periprosthetic joint infections (PJI). The purpose of this study was to investigate the outcomes of patients who underwent MKA for PJI with a single technique and determine if specific factors are associated with MKA failure.

**Methods:**

This was a retrospective review of 81 patients who underwent MKA at a single institution. Knee Society Scores were recorded before MKA and at the final follow-up (mean 52 months). Poisson regression was used to calculate rate ratios for MKA failure secondary to infection.

**Results:**

The mean patient age was 67 years; most patients were McPherson B hosts (56.8%) and had type 3 extremities (53.1%), and all had a type III infection (chronic, >4 wks). Forty-six percent of patients had a prior explantation (59.5% failed 2-stage, 40.5% failed spacer). *Staphylococcus epidermidis* and *Staphylococcus aureus* were the most common organisms, 22.2% and 18.5%, respectively. Thirty percent of patients had at least one reoperation, excluding reimplantation (14.8% irrigation and debridement/wound closure, 9.9% MKA exchange, and 7.4% amputation). Of 82.7% of MKA patients with no evidence of infection, 82.1% (56 patients) underwent reimplantation endoprosthetic reconstruction, and 67.3% of these remained infection-free at the final follow-up.

**Discussion:**

MKA is a salvage option for challenging PJI cases that may serve as definitive surgical management or as a bridge to endoprosthetic reconstruction for patients who have failed prior infection control procedures.

## Introduction

Periprosthetic joint infection (PJI) remains a devastating complication of total knee arthroplasty (TKA). PJI affects approximately 1%-2% of primary TKA, and this risk increases to 3%-5% with revision surgery [1,2]. PJI is the most common reason for revision TKA, accounting for 25.2% of revision cases [3]. It is associated with a significant decrease in patient quality of life and increase in morbidity and mortality. When compared with aseptic revision knee arthroplasty, Zmistowski et al found that revision surgery for PJI was associated with significantly higher mortality rates at all time points analyzed, including 10.6% vs 2.0% at 1 year and 25.9% vs 12.9% at 5 years [3,4]. Furthermore, PJI poses a huge financial burden to the United States health-care system, with a projected annual cost of $1.62 billion dollars in 2020 [[5], [6], [7]].

The ability to eradicate a PJI is multifactorial and depends on several factors including the infecting organism type, chronicity of infection, as well as host-specific factors. The McPherson staging system is a validated system that can be used to better define PJI. It includes 3 categories: infection type (acute <4 weeks vs chronic ≥4 weeks), systemic host grade (defines degree of host immune system compromise based on certain comorbidities), and local extremity grade (stratification based on soft tissue, vascular, and osseous integrity) [8,9].

Treatment of chronic PJI using a two-stage surgical protocol is the current gold-standard in the United States [[10], [11], [12], [13]]. Despite the growing interest in managing PJI with single-stage revision, two-stage surgery remains the more well-established modality for treating hosts with poorer bone quality and more virulent organisms [14]. Knee spacer options can be classified as articulating or static. No significant difference in infection eradication rates has been observed between the two treatment options; however, one may be more appropriate than the other depending on infection chronicity, number of prior surgeries, compromising host factors, and the condition of the surrounding soft tissues [[15], [16], [17], [18], [19], [20], [21]]. Modular knee arthrodesis (MKA) is a static spacer construct and is also a salvage surgical option as an alternative to an above-knee amputation (AKA) in patients who have failed multiple debridements or multiple attempts at two-stage reimplantation [1,[22], [23], [24], [25]]. The currently available comparative outcomes studies of knee fusion vs AKA consist of small numbers of patients in either cohort with multiple surgical fusion techniques. These studies show mixed functional outcomes when comparing the two groups; however, as shown by Chen et al., knee fusion is associated with superior mental well-being presumably associated with limb preservation [26].

At our institution, an antibiotic cement-coated modular MKA prosthesis has been used for knee fusion in patients with complex chronic PJI in the following settings: failed 2-stage reimplantation, persistent PJI despite an antibiotic spacer, PJI with significant bone loss or soft-tissue compromise such as resulting from severe osteolysis, periprosthetic fracture, or soft-tissue loss. This protocol is aimed at delivering a high concentration of antibiotics locally, maintaining the joint space, preserving the soft tissues, and allowing early mobilization of patients.

There is a paucity of literature on outcomes of knee MKA as a salvage treatment in the setting of complex PJI cases. The purpose of this study is to report on the clinical and functional outcomes of MKA at our institution in the context of complex PJI.

## Material and methods

**Figure 1:**
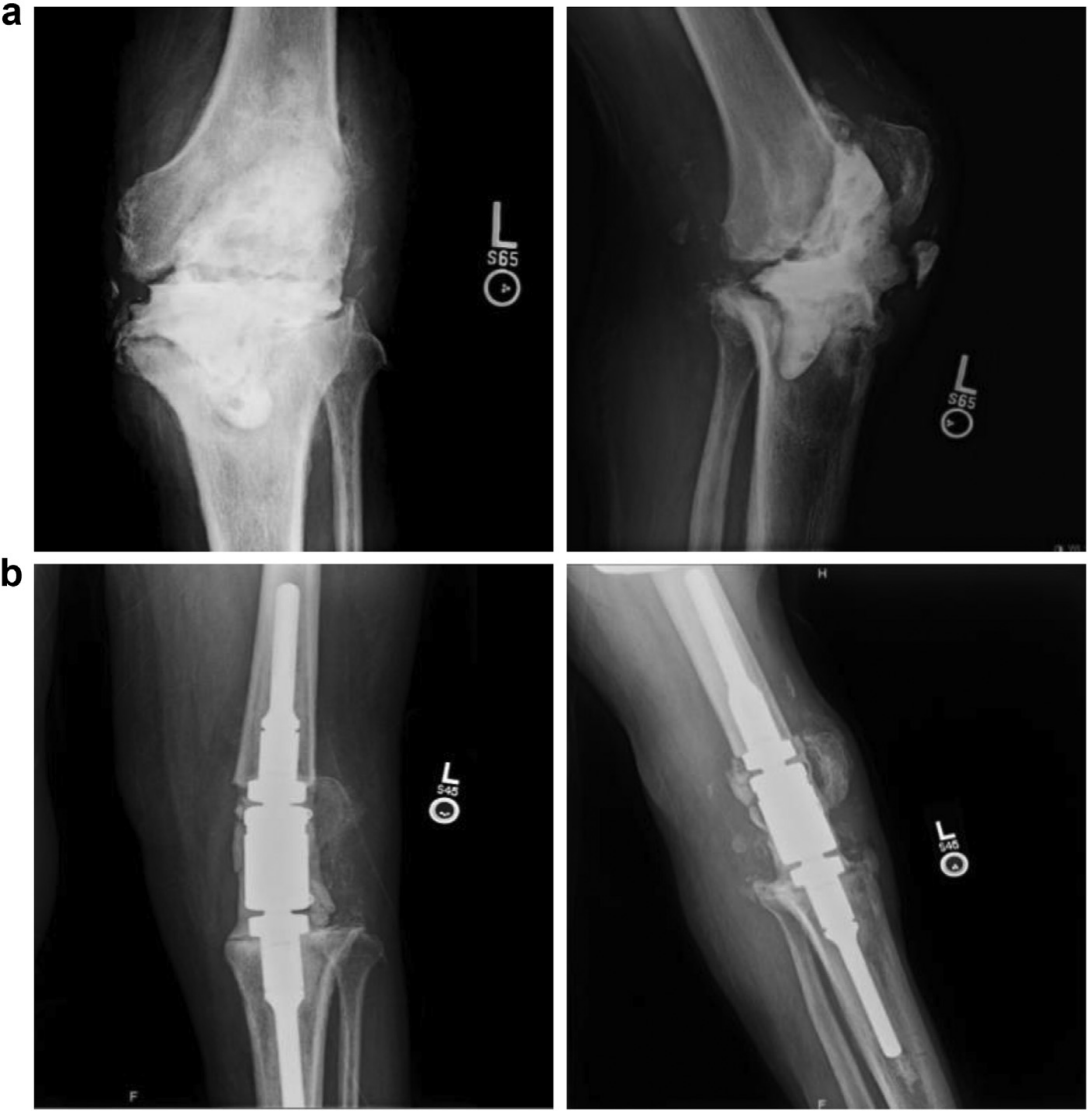
Radiographs of a 67-year-old male with (a) persistent *Pseudomonas aeruginosa* periprosthetic joint infection despite explantation and articulating spacer placement. (b) The Patient underwent knee fusion with the modular endofusion system and has no evidence of infection at the latest follow-up.

This was a retrospective review of 81 patients who underwent MKA at a single institution with a single surgeon in a high-volume revision arthroplasty practice, from 1998 to 2019, with a mean of 52 months of follow-up. This study was approved by our institutional review board. Patients were included if they underwent MKA for PJI and were excluded if MKA was performed for oncological purposes. Musculoskeletal Infection Society criteria were used to diagnose PJI. MKA was performed using a cemented modular arthrodesis system (OSS Modular Arthrodesis System, Zimmer Biomet, Warsaw, IN) (Fig. 1). Before MKA, extensive soft-tissue and osseous debridements were performed. The distal femur and proximal tibia were resected as needed based on the intraoperative appearance, and the femoral and tibial intramedullary canals were prepared with flexible reamers. Thorough irrigation of the surgical site was performed with a bacitracin irrigation solution. The MKA cement technique uses vancomycin 5 g, tobramycin 3.6 g, 1-cc methylene blue, and 9-cc sterile saline per bag of medium viscosity cement. Antibiotic cement was also used to coat any exposed intra-articular metal. Every case in this study involved infection, and for our purposes, the construct was not intended to result in primary bone fusion. The goal of the endofusion device was to maintain tissue tension and integrity, as well as leg length. As such, the intra-articular space was spanned by endofusion intercalary segments and a clamshell connector. These were all covered with antibiotic-loaded cement. The clamshell connector measures 6.5 cm in length, thus the total amount of bone resection is 7 cm. To assist in pressurization, a cement restrictor was inserted into the intramedullary canal before cementation. All patients were placed on IV antibiotics postoperatively, which were adjusted as needed based on medical comorbidities (eg, chronic kidney disease, allergies, and so on) and final intraoperative cultures.

**Figure 2:**
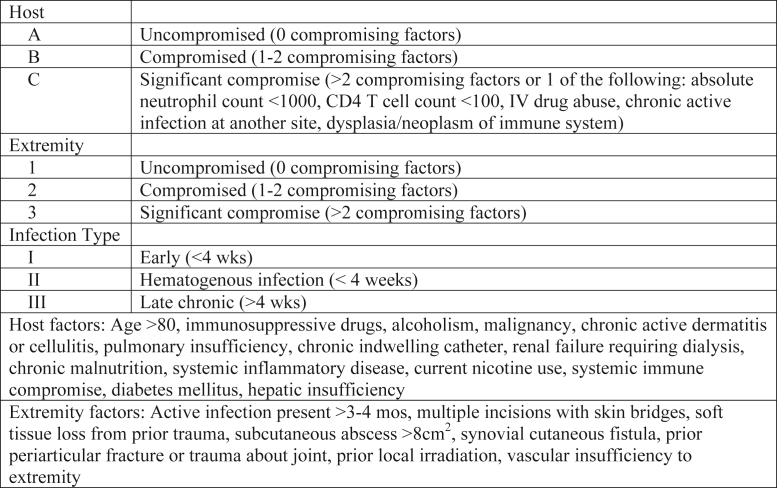
McPherson host staging system.

Patient characteristics included patient age, sex, time from index arthroplasty surgery to MKA, and history of failure of prior explantation with an articulating antibiotic spacer or failed 2-stage reconstruction secondary to recurrent PJI. The McPherson host staging system was used to further characterize patients (Fig. 2). Infecting organism information was obtained from knee aspiration before MKA and from intraoperative cultures at the time of MKA.

The primary outcome of interest was MKA failure as defined by the reinfection rate. Reinfection was measured by positive aspiration results and/or surgical specimen during revision surgery after index MKA. A cleared infection after MKA was defined as those who had culture-negative aspiration results and/or surgical specimen results (3 surgical specimens sent) along with normal inflammatory markers.

Medical complications were documented during the index MKA hospitalization and up to 90 days postoperatively. Medical complications included pneumonia, urinary tract infection, deep venous thrombosis, pulmonary embolism, myocardial infarction, cardiac arrest, cerebrovascular accident, or acute renal failure. Surgical complications were tracked throughout the study period. Surgical complications included wound dehiscence, superficial surgical site infection, deep surgical site infection, periprosthetic fracture, aseptic loosening, and mechanical failure of the MKA device.

Reoperation was also documented, including irrigation and debridement/wound closure, periprosthetic fracture fixation, AKA, MKA exchange, and reimplantation. MKA exchange was performed for persistent infection in patients who refused AKA or in patients who developed aseptic loosening who were not candidates for reimplantation.

Knee Society Scores (KSS) were recorded before MKA and at the most recent follow-up period.

SAS 9.3 was used for the statistical analysis in this study. Poisson regression was used to calculate rate ratios (RRs) for MKA failure based on infecting organism and McPherson host type. We chose these variables as we suspected they would highly influence infection recurrence. Statistical significance was defined as *P* < .05.

## Results

Eighty-one patients were included in the study, 51.9% were female, and the mean patient age at the time of MKA was 67 years. Mean time from index primary TKA surgery to MKA was 25 months. Forty-six percent of patients had a prior explantation; of these, 59.4% with a failed 2-stage and 40.5% with persistent infection of an articulating spacer. Most patients were McPherson classification host grade B (56.8%) with a local extremity score of 3 (53.1%), and all patients had a type III infection (chronic, >4 wks) (Table 1).

**Table 1:** Patient characteristics.

Age (y)	67 ± 7.9 (45 – 84)
Sex (%)	
Female	51.9
Male	48.1
Time to fusion (mo)	25 ± 31.9 (1 – 154)
Prior explantation (%)	45.6
Failed 2-stage	59.4
Failed articulating spacer	40.5
McPherson host staging system (%)	
Host	
A	13.6
B	56.8
C	29.6
Extremity	
1	1.23
2	45.7
3	53.1
Infection type	
I	0
II	0
III	100

*Staphylococcus epidermidis* was the most common infecting organism (22.2% of cases), followed by 18.5% with *Staphylococcus aureus* (60% methicillin-sensitive *Staphylococcus aureus* and 40% methicillin-resistant *Staphylococcus aureus*). Approximately 11% of patients had a multiorganism infection, and another 11.1% had a culture-negative diagnosis (Table 2).

**Table 2:** Organism characteristics.

Organism	%
*Staphylococcus aureus*	18.5
MSSA	11.1
MRSA	7.4
*Staphylococcus epidermidis*	22.2
*Enterococcus faecalis*	7.4
*Escherichia coli*	4.9
*Pseudomonas aeruginosa*	2.5
Candida	4.9
Other organisms	17.5
Multiple organisms	11.1
Culture negative	11.1

MSSA, methicillin-sensitive Staphylococcus aureus; MRSA, methicillin-resistant Staphylococcus aureus.

After MKA, there was an 8.6% index hospitalization complication rate and 29.6% overall complication rate, excluding reinfection. Of the overall complications, 33.3% were medical, and 66.6% were surgical. Seventeen percent of patients had a persistent infection or reinfection after MKA. There were a total of 32 reoperations in 24 patients, excluding reimplantation arthroplasty (Table 3). The most common reason for repeat surgery was for irrigation and debridement and wound closure (14.8%). Of the patients with persistent infection or reinfection of the MKA, 9.9% underwent repeat MKA, and 7.4% underwent AKA. There were no MKA exchanges for aseptic loosening. Of the 82.7% of patients with no evidence of infection, 82.1% (56 patients or 67.9% of the entire cohort) elected to proceed with reimplantation endoprosthetic reconstruction. The mean time between MKA and reconstruction was 220 days, and the median was 217 days (range 77 – 350 days). We assessed extensor mechanism integrity at the time of reconstruction and found that 46.4% (26 patients) had an intact extensor mechanism and 53.6% (30 patients) had a deficiency. Three of these 56 patients required long-term suppressive antibiotics after endoprosthetic reconstruction while 67.3% remained infection-free at a final mean 41-month reimplantation follow-up (Table 3). At the final follow-up, 11 patients (13.5%) had retained an MKA and were infection free.

**Table 3:** Patient outcomes.

Complications and reoperation	N	%
Index hospitalization complications	7	8.6
Overall complications (excluding reinfection)	24	29.6
Medical	8	33.3
Surgical	16	66.6
Persistent infection/reinfection	14	17.3
Reoperation (excluding endoprosthesis conversion)	24	29.6
Amputation	6	7.4
Fusion exchange	8	9.9
Irrigation and debridement, wound closure	12	14.8
Fracture fixation	6	7.4
No evidence of reinfection	67	82.7
Conversion to endoprosthesis	55	82.1
Infection-free survival of reimplantation	37	67.3

Although the RR for MKA failure secondary to infection was higher for certain organisms, this RR did not reach statistical significance. There was no statistically significant association between MKA reinfection and index infecting organism (Table 4). There were no MKA failures secondary to reinfection in McPherson grade A hosts or those with a McPherson type 1 extremity. There was no significant difference in MKA/endoprosthetic reinfection failure between grade B and C hosts and those with a type 2 or 3 extremity (Table 5). Similarly, all patients with endoprosthetic reconstructions who became reinfected were McPherson grade B or C and had a type 2 or 3 local extremity. Clinical KSS after MKA were lower than scores before MKA (31 ± 5.7 to 23 ± 3.6, *P* < .05). There was no significant difference in functional KSS before and after MKA (15 ± 4.3 to 14 ± 4.3, *P* > .05) (Table 6).

**Table 4:** Endofusion reinfection risk by organism.

Organism	RR (95% CI)	*P* value
MSSA	1.56 (0.10-24.87)	.7547
MRSA	2.33 (0.15-37.30)	.5491
*Staphylococcus epidermidis*	3.11 (0.35-27.84)	.3100
*Enterococcus faecalis*	7.00 (0.73-67.30)	.0919
*Escherichia coli*	3.50 (0.22-55.96)	.3757
*Pseudomonas aeruginosa*	0	
Candida	3.50 (0.22-55.96)	.3757
Other organisms	1	
Multiple organisms	1.56 (0.10-24.87)	.7547
Culture negative	3.11 (0.28-34.31)	.3541
Organism type		
Gram positive	1.62 (0.21-12.79)	.6472
Culture negative	2.00 (0.18-22.06)	.5714
Gram negative	1.00	
Multi	1.00 (0.06-15.99)	1.000
Fungal	2.25 (0.14-35.97)	.5664

MSSA, methicillin-sensitive S. aureus; MRSA, methicillin-resistant S. aureus.

**Table 5:** Endofusion reinfection risk by McPherson host.

Grade	Failure (%)	RR (95% CI)	*P* value
A	0		
B	19.6 (6.8, 32.4)	1	
C	20.8 (1.3, 37.8)	1.06 (0.36, 3.18)	.9104
Extremity			
1	0		
2	13.5 (1.7, 25.4)	1	
3	23.3 (8.8, 37.7)	1.72 (0.59, 5.03)	.3216

**Table 6:** Knee Society Scores.

Prefusion		
Clinical	31 ± 5.7 (8 – 65)	
Functional	15 ± 4.3 (-20 – 60)	
Postfusion		
Clinical	23 ± 3.6 (0 – 65)	*P* < .05
Functional	14 ± 4.1 (-20 – 60)	*P* > .05

## Discussion

In this study, we describe the outcomes of knee fusion using a single MKA technique to treat challenging PJI cases resistant to prior attempts at eradication and/or with significant bone loss and soft-tissue compromise. Two-third of the patients eventually underwent reimplantation endoprosthetic reconstruction, and the majority remained infection-free at the final follow-up. These results are especially promising given that almost half the cohort who underwent MKA had previously undergone a failed 2-stage exchange or had a persistent infection of an antibiotic cement spacer.

Although MKA provides a salvage option for patients who have failed multiple infection control methods, our findings suggests that the results of this knee fusion procedure are not well tolerated. Patient knee function was essentially unchanged based on KSS scores after MKA but did get worse clinically. Given that all these patients were seeking treatment for a chronically infected knee, which had failed prior articulating spacer or a 2-stage revision TKA, knee function scores were relatively low at baseline and did not improve after surgery. While it is helpful to know that there was no functional decline after MKA despite losing dynamic knee mobility, it may be that living with an MKA is no better than living with a chronically infected prosthesis or is at least poorly tolerated long term. In fact, over 80% of our patients elected to undergo reimplantation and endoprosthetic reconstruction after MKA. Unfortunately, we did not evaluate patient quality of life via objective measures in our study and, therefore, cannot directly comment on overall patient satisfaction beyond the KSS scores and the observation that most patients eventually sought reconstruction.

Fortunately, MKA does appear at least to have a role as a static antibiotic spacer in patients who have failed other infection-control procedures. Our patients experienced a high eradication rate, and the majority were eventually able to undergo reimplantation and remained infection free at the final follow-up. Also, for the smaller percentage of patients who are interested in limb salvage, but may not be candidates for reconstruction, MKA offers a viable alternative to AKA with the provision that it may not provide much functional improvement. Further research is needed to compare the functional outcomes of MKA patients with those of patients who undergo AKA. The comparisons in the literature are somewhat outdated and may not reflect changes in surgical technique, postoperative recovery protocols, or prosthetic options [22,23,25,27].

Gramlich et al. described similar results in their cohort of 52 MKA patients with a PJI remission rate of 88.5% [28]. They also had a comparable rate of amputation after MKA (9.6%). Their study, which compared MKA to a matched control group of patients with a rotating hinged revision TKA, found that KSS functional scores were less in the MKA group. Not surprisingly, quality of life outcomes were also inferior compared with those in patients who underwent hinged revision TKA.

With the high rate of conversion from MKA to reimplantation in our study, our results warrant comparison to those undergoing cemented antibiotic static spacer placement after failed two-stage revision TKA or failed articulating spacer placement. To our knowledge, there are no direct comparison of the MKA device to other static spacer implants after a failed two-stage exchange or uncleared articulating spacer infection. Reinfection rate after initial two-stage reimplantation ranges from 80% to 90% [[29], [30], [31]]. However, infection eradication with each successive attempt at reimplantation worsens. Outcomes are less promising after two 2-stage revision arthroplasties with remission rates in the 50%-60% range [32,33]. A meta-analysis of patients with a failed 2-stage revision showed a lower risk of failure with knee arthrodesis than with repeat 2-stage revision. The reinfection rates in our article (67.3% reimplantation infection-free survival) are somewhat better than those in patients who have undergone multiple two-stage exchanges. MKA may, therefore, be suitable in these patients seeking limb salvage to leave open the possibility of eventual reconstruction after infection eradication.

There are several limitations to this study. The first is that this study was retrospective, and patients were not randomized. Second, patients underwent only one type of knee fusion construct, with a modular endofusion device, and the outcomes of this study may not be applicable to other types of knee fusion constructs such as plate fixation, intramedullary nailing, or external fixation/Ilizarov/Taylor Spatial Frame methods [34]. Another weakness of this study is the small sample size that did not allow for differences between Mcpherson groups or infecting bacteria to be used. Still, this is the largest reported series to date using a MKA system for patients with recurrent PJI.

One of the main advantages of a modular knee endofusion constructs such as the one used in this study is that, unlike some of the other knee fusion techniques, there is immediate direct fixation via cement interdigitation, and there is no need to rely on the host to provide bony union at the fusion site. This is particularly advantageous in PJI situations where infection may interfere with bone healing or there is significant bone loss either through severe osteolysis or concomitant periprosthetic fracture [35]. Besides the ease in technique, using a short modular implant compared with the long nail constructs described in other knee fusion studies avoids potentially seeding the hip joint in cases of PJI. Another advantage of this type of construct is that it also allows for the elution of high levels of antibiotics from the cement across an extensive surface area, which may contribute to the high eradication rate after MKA seen in this study. The main concern with using a cemented endofusion construct for a long term is the risk of aseptic loosening, but in our study, there were no MKA exchanges for aseptic loosening and all were performed for persistent infection.

In conclusion, this study presents the outcomes of one of the largest single institution cohorts of patients who have undergone cemented modular endofusion specifically for PJI. While MKA may present a salvage option in extremely challenging knee PJI cases resistant to other modes of treatment, it may also serve as a static spacer with the eventual goal of endoprosthetic reconstruction after infection eradication. Further research is needed to assess the clinical and functional outcomes of MKA compared with other treatment options for patients with recurrent PJI (eg, amputation).

## Conflicts of interest

E. J. McPherson receives royalties from Zimmer-Biomet; is in the speakers’ bureaue of or gave paid presentations for Austin Medical Ventures and Zimmer-Biomet; is a paid consultant for Zimmer-Biomet; is in the editorial/governing board of Reconstructive Review.

## Appendix A Supplementary data

Conflict of Interest Statement for McPherson

Conflict of Interest Statement for Chowdhry

Conflict of Interest Statement for Dipane

Conflict of Interest Statement for Stavrakis

Conflict of Interest Statement for Mayer

Conflict of Interest Statement for Devena
